# Soil moisture and nitrate-nitrogen dynamics and economic yield in the greenhouse cultivation of tomato and cucumber under negative pressure irrigation in the North China Plain

**DOI:** 10.1038/s41598-019-38695-4

**Published:** 2019-03-14

**Authors:** Yinkun Li, Xuzhang Xue, Wenzhong Guo, Lichun Wang, Minjie Duan, Hong Chen, Fei Chen

**Affiliations:** 1 Beijing Research Center of Intelligent Equipment for Agriculture, Beijing Academy of Agriculture and Forestry Sciences, Beijing, 100097 China; 2 Key Laboratory of Urban Agriculture (North China), Ministry of Agriculture, Beijing, 100097 China; 3 Beijing Institute of Landscape Architecture, Beijing, 100102 China; 4 Beijing Key Laboratory of Ecological Function Assessment and Regulation Technology of Green Space, Beijing, 100102 China

## Abstract

A field experiment was carried out for two years to investigate the benefits of negative pressure water supply on surface soil water content, nitrate-nitrogen (NO_3_^−^-N) distribution in the soil profile, economic yield and water and fertilizer use efficiency of tomato and cucumber under greenhouse cultivation in the North China Plain. The experiment included two irrigation treatments: drip irrigation with nutrient solution (DIN) and negative pressure irrigation with nutrient solution (NIN). The results showed that the NIN treatment had a relatively stable soil moisture (about 87% of field capacity), and the fluctuation of soil water content in the 0–20 cm soil layer was 20.6%–25.0% during the experiment period in 2014–2015, which was less than the range of 19.2%–28.1% in the DIN treatment. In both the DIN and NIN treatments, the NO_3_^−^-N at the end of the four growing seasons was mainly distributed in the 0–40 cm soil layer and showed a gradually increasing trend as the number of cultivation years increased. Compared with the DIN treatment, the NO_3_^−^-N content in the 0–60 cm layer of the NIN treatment was significantly decreased by 19.7%–28.0% after the fourth growing season. The NIN treatment produced the highest economic yield with lower water and nutrient input than the DIN treatment, however, no significant difference was observed in tomato and cucumber yield in the two years. Average irrigation water use efficiency (WUE_i_) and partial factor productivity of fertilizer (PFP_f_) over the study period were all significantly improved under the NIN treatment relative to the DIN treatment, with increases of 26.2% and 25.7% (P < 0.05), respectively. Negative pressure water supply not only maintained a high fruit yield, but significantly increased WUE_i_ and PFP_f_, indicating a great advantage in water and fertilizer saving compared with drip irrigation.

## Introduction

Greenhouse vegetable production has developed rapidly in China because of the relatively high prices of vegetables compared with cereal crops, and the cultivated area of greenhouse vegetables reached 4.0 million ha in 2015^1^, accounting for 18.2% of the total national vegetable planting area^2^. Both water and fertilizer are essential factors for vegetable growth and influence fruit yield, but excessive water and fertilizer input is a common practice by farmers in China to ensure high yields^3,4^. For instance, it has been shown that the irrigation water rate of fruit vegetables is 750–1050 mm per year^5^, and the fertilizer input of more than 1000 kg N ha^−1^ per growing season far exceeded vegetable demand in a Beijing suburb^6^. Consequently, the overuse of water and fertilizer has resulted in negative environmental impacts such as decreased soil pH and enhanced soil salinization and groundwater pollution^7,8^.

Irrigation is the primary factor in improving vegetable yield, and irrigation methods with a contribution on saving water and fertilizer should be encouraged^9,10^. Flooding and furrow irrigation have been the major types of irrigation for a long time in greenhouse vegetable production in China because of their low cost^11,12^. However, the conventional irrigation method usually far exceeds the crop’s needs or the soil’s water-holding capacity and leads to low water use efficiency and wasted nitrogen (N)^13,14^. Drip irrigation is an effective way to supply water and fertilizer to the root zone and not only saves water but can also increase fruit yield. Fan *et al*.^14^ resulted showed that compared with conventional flooding irrigation, fertilizer N and water inputs were reduced by 78% and 43% with drip irrigation, respectively. Mahajan *et al*.^15^ also reported that drip irrigation can save 48.1% of irrigation water and result in a 51.7% higher fruit yield compared with surface irrigation for greenhouses. Drip irrigation with its characteristic of low discharge rate reduces crop evapotranspiration and deep percolation, but has been shown to increase tomato fruit number and fruit size, thus improving water use efficiency^16^. The irrigation methods discussed above are surface irrigation techniques, which are driven by positive pressure. Although those irrigation methods offer certain advantages such as simple operation and wide applicability, the irrigation is scheduled mainly according to conventional experience, which may induce water wastage, nutrient loss and poor fruit quality^17^.

Negative pressure irrigation is a new irrigation technique that has received research attention in recent years. Unlike the positive pressure irrigation techniques, negative pressure irrigation systems supply water with the pressure in negative values, the principle of controlling water release in this system is based on the soil water potential difference^18^. Moreover, the irrigation emitter of the negative pressure irrigation system is buried in the soil, which could reduce water losses from soil evaporation and deep percolation. Automatic irrigation can be realized and the labor cost is correspondingly greatly reduced with this technique^19^. Thus, irrigation can be scheduled according to crop requirements when using negative pressure irrigation system. Li *et al*.^20^ showed that water consumption mainly from plant growth demand under negative pressure irrigation conditions, and there were no significant difference between crop evapotranspiration and irrigation amount. With the use of negative pressure irrigation, the soil water content was stable during the crop growing season, and a variable coefficient of soil moisture of less than 0.1 was observed during the cucumber growth period based on a pot experiment in a greenhouse^21^. Zhao *et al*.^22^ also reported that the gravimetric soil water contents in sandy loam soil were 28.5%, 22.5%, 18.1% and 15.4% when the water pressure was 0, −5, −10 and −15 kPa, respectively, and water supply pressure from −10 to −5 kPa was the optimum for bok choy.

Currently, a number of studies have been conducted on the impact of negative pressure water supply on vegetable growth and water use in soilless cultivation and potted systems^21,23^. However, very few studies have focused on the effect of continuous negative pressure irrigation on the annual variation of soil moisture in soil vegetable cultivation under greenhouse conditions, and there is no report on the nitrate-nitrogen (NO_3_^−^-N) distribution along the soil profile of negative pressure irrigation in greenhouse vegetable production. Therefore, we conducted a field plot experiment to investigate the influence of negative pressure water supply and drip irrigation treatments on surface soil moisture variation and NO_3_^−^-N distribution in the soil profile. The differences in economic yield, irrigation water and fertilizer use efficiency were analyzed between the two irrigation methods. In addition, we investigated the temporal variation of soil moisture and NO_3_^−^-N, as well as the irrigation water and fertilizer use efficiency under the negative pressure conditions to reveal the mechanisms that explain why negative pressure irrigation can save water and fertilizer compared with drip irrigation.

## Materials and Methods

### Experimental site

The experiment was carried out in a greenhouse from March 2014 to January 2016 at the National Experiment Station for Precision Agriculture (40°10′43″N, 116°26′39″E), located in Xiaotangshan Beijing, China. The experimental region has a temperate monsoon climate. The annual mean temperature and precipitation are 11.8 °C and 550.3 mm. The greenhouse had silt loam soil (sand, 25%; silt, 69%; clay, 6%) with a pH of 6.75, field capacity (FC) of 26.3% (weight basis) and bulk density of 1.39 g cm^−3^. The organic matter, total N, available phosphorus (P_2_O_5_) and potassium (K_2_O) contents in the 0–20 cm soil layer before transplantation were 23.3, 1.57, 0.10 and 0.16 g kg^−1^, respectively.

### Experimental design

The experimental area in the greenhouse was 28 m × 7.5 m. The experiment was laid out in a randomized block design with two treatments and three replications. The two treatments were drip irrigation with nutrient solution (DIN) and negative pressure irrigation with nutrient solution (NIN). The experimental plots were 5.0 m × 1.4 m, and an impermeable PVC board was embedded vertically in the soil to a depth of 60 cm between plots to prevent lateral infiltration of water.

**Figure 1:**
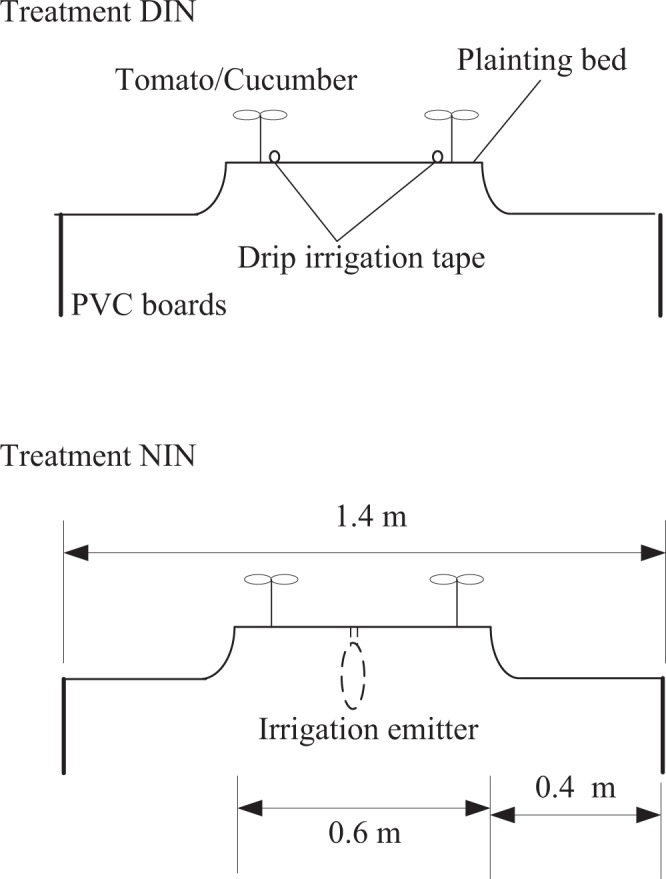
Layout of the drip irrigation tape and irrigation emitter in the experimental plot.

The experiment period consisted of four growth cycles including early spring 2014 (2014 ES, March–July), autumn–winter 2014 (2014AW, August–December), early spring 2015 (2015ES, March–July) and autumn–winter seasons 2015 (2015AW, September–January the following year). The varieties of tomato and cucumber used in the experiment were “Xianke 8” and “Zhongnong No. 26”, respectively. Tomatoes were planted in 2014ES, 2014AW and 2015AW, respectively, and cucumbers were planted only in the 2015ES season. In this experiment, planting beds were prepared 0.8 m apart were 0.6 m wide at the top (Fig. 1). The tomato and cucumber seedlings were transplanted at the 2–3 leaf stage along the edge of raised beds that were 0.6 m wide and 0.2 m high in two rows that were 0.4–0.5 m apart with 0.35 m between each plants. In the DIN treatment, irrigation was carried out in the form of gravity drip irrigation with two drip irrigation tapes laid in the center of every ridge. The dripper spacing was 0.15 m, dripper flow rate was 1.38 L h^−1^ and every crop root had a dripper for water supply.

**Figure 2:**
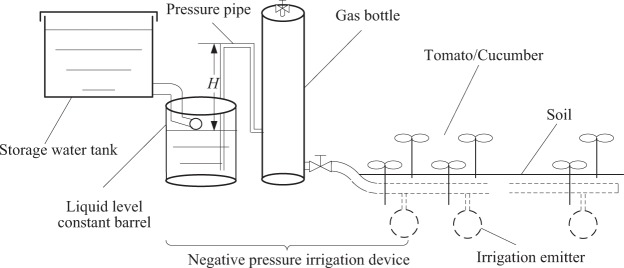
Structure of the negative pressure irrigation system.

In the NIN treatment, the negative pressure irrigation system was established with negative pressure irrigation device, and integration of water and fertilizer can be achieved in this system (patented by the Beijing Research Center of Intelligent Equipment for Agriculture (patent no. 201510036539.7)). The structure of the negative pressure irrigation system is illustrated in Fig. 2. The irrigation system consists of five parts: storage water tank, liquid level constant barrel, pressure pipe, gas bottle and irrigation emitter (Fig. 2). The latter consists of a 0.2 m diameters porous ceramic plate and was buried at 0.25 m soil depth at equal intervals of 0.35 m in the current study. The tomato/cucumber seedings were planted on each side of the irrigation emitter at a distance of 0.2 m from the emitter (Fig. 1). When the negative pressure irrigation system is running, crop roots proceed to absorb water from the soil, resulting in release of irrigation water from the irrigation emitter into the soil under the action of soil water potential difference. In this case, the pressure in the gas bottle will reduce under the decline of the liquid level. With the difference of atmospheric pressure, the irrigation water in the liquid level constant barrel enters the gas bottle through the pressure pipe. In the liquid level constant barrel, a float valve keeps water at the same level, and the water is supplied through the liquid storage barrel. Finally, the irrigation rate can be obtained by recording the water-level difference in the liquid storage barrel. The detailed working principles of this system are described in Li *et al*.^24^. In the current study, we set the suction at −5 kPa according to previous studies^23^. The irrigation amounts of treatment DIN and NIN are listed in Table 1.

**Table 1:** Nutrient (N + P_2_O_5_ + K_2_O) rates and irrigation amount in the experiment in 2014 and 2015.

Cropping season	Treatment	N (kg ha^−1^)	P_2_O_5_ (kg ha^−1^)	K_2_O (kg ha^−1^)	Irrigation (mm)
2014 ES	DIN	422.8	159.9	629.6	336.6
	NIN	375.2	141.9	558.7	298.7
2014 AW	DIN	356.0	134.7	530.2	283.5
	NIN	269.7	102.0	401.5	214.9
2015 ES	DIN	368.9	109.5	433.9	308.8
	NIN	313.4	93.1	368.6	262.3
2015 AW	DIN	361.6	136.7	538.4	287.8
	NIN	287.7	108.8	428.5	229.1

NIN: negative pressure irrigation with nutrient solution. DIN: drip irrigation with nutrient solution.

In the current study, Japan Yamazaki tomato and cucumber nutrient solution formula were used for tomato and cucumber, respectively. The fertilizers included calcium nitrate tetrahydrate (N, 17.1%; Ca, 24.4%), potassium nitrate (K_2_O, 46.3%; N, 13.8%), ammonium dihydrogen phosphate (P_2_O_5_, 61.7%; N, 12.2%) and magnesium sulfate heptahydrate (Mg, 9.8%; S, 13.0%), and were applied with 1 L of irrigation water at rates of 354, 404, 77 and 246 mg for tomato, and 413, 304, 58 and 242 mg for cucumber, respectively. The nutrient solution was added to the irrigation barrel every time that irrigation was applied. The fertilizer amount (NPK) was calculated according to the irrigation rates and nutrient concentrations. For the NIN treatment, the irrigation amount reduced by 11.3%–24.3%, and the nutrient input (N + P_2_O_5_ + K_2_O) also decreased by 11.3%–24.3% compared with DIN.

### Measurements

Soil samples were collected for the soil layers of 0–20 cm by mixing three soil cores (3 mm) from each plot, at the stages of transplanting, seeding, flowering, fruit-set, picking and harvesting, and soil water content was measured by the oven drying method. After crops were harvested in each of the four seasons, soil samples were also taken from each plot at 20 cm intervals from 0–100 cm depth. Collected samples were divided into two subsamples after sieving to 2 mm; one subsample was used to measure soil water content following the standard gravimetric method, and the other was used to determine the soil NO_3_^−^-N content. The samples were extracted with 2 M KCl at the ratio of 1:10 (soil: solution, w/v) for 1 h. The soil NO_3_^−^-N concentration was measured using a continuous flowing analyzer (SEAL AutoAnalyzer 3, Norderstedt, Germany).

Soil water content was also measured 1 day before irrigation in the DIN treatment, and tomato plants were irrigated to 90% of field capacity (*θ*) when mean soil water content in the main root zone (0.3 m) was depleted to 70% of *θ*. The water amount in each drip irrigation event (DIN treatment) was calculated as^25^:

$${I}_{{\rm{w}}}=1000\times \rho \times (0.9\theta -{\theta }_{i})\times h$$

where *I*_w_ is the amount of irrigation water (mm); *ρ* is the soil bulk density (g cm^−3^), *θ* is the soil field capacity (g g^−1^), *θ*_i_ is the actual soil moisture content before irrigation (g g^−1^), 0.9 was the irrigation coefficient (90% of field capacity) and *h* is the planned moisture layer depth (0.3 m).

Crop evapotranspiration (ET, mm) was calculated by water balance equation^26^. There was no precipitation in the greenhouse, and surface runoff and deep percolation can be neglected under drip irrigation and negative irrigation^20,25^, thus ET can be estimated by:

$${\rm{ET}}={I}_{{\rm{w}}}+{\rm{\Delta }}W$$

where ΔW is the change in soil water storage (mm).

Total economic yield was measured for whole tomato/cucumber growth cycle in each plot and translated into fruit yield weight per hectare. The ratio of yield to water supply for treatment DIN and NIN was referred to as irrigation water use efficiency (WUE_i_, t ha^−1^mm^−1^):

$${{\rm{WUE}}}_{{\rm{i}}}=Y/{I}_{{\rm{r}}}$$

where *Y* and *I*_r_ represent the total economic yield (t ha^−1^) and the total irrigation amount (mm), respectively. The ratio of yield to chemical fertilizer (N + P_2_O_5_ + K_2_O, kg ha^−1^) is referred to as partial factor productivity of applied fertilizer (PFP_f_, kg kg^−1^):

$${{\rm{P}}{\rm{F}}{\rm{P}}}_{{\rm{f}}}=Y/F\times 1000$$

where *F* is the amount of applied fertilizer (N + P_2_O_5_ + K_2_O, kg ha^−1^).

### Statistical analysis

Statistical analysis of variance (ANOVA) was done using SAS software version 9.1. Treatment means were separated using the least significant difference (LSD) test at P < 0.05. The figures were prepared using SigmaPlot software version 12.0.

## Results

### Microclimate inside and outside the greenhouse

**Figure 3:**
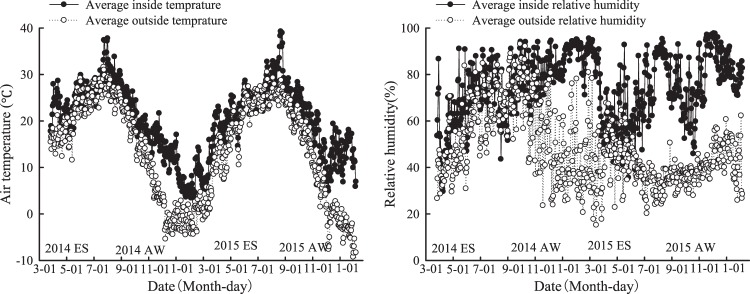
Microclimate variation of inside and outside the greenhouse during the experiment in 2014–2015.

Daily average air temperature and relative humidity inside and outside the greenhouse are shown in Fig. 3. The variation of air temperature inside the greenhouse was similar to that outside the greenhouse, and the temperature tended to increase in the ES season and gradually decrease in the AW season. Mean daily temperature were 24.6, 20.8, 22.4 and 17.1 °C inside the greenhouse, and 21.6, 13.8, 19.1 and 7.11 °C outside the greenhouse during the 2014 ES, 2014AW, 2015 ES and 2015AW seasons, respectively.

The variation of relative humidity was also similar inside and outside the greenhouse during the 2014ES season, and the mean daily relative humidity was 65.0% and 52.5% inside and outside the greenhouse, respectively. However, there was a considerable difference in relative humidity inside and outside the greenhouse during the AW season; mean daily relative humidity was 76.6% and 78.8% inside the greenhouse and 42.7% and 41.2% outside the greenhouse in the 2014AW and 2015AW seasons, respectively.

### Changes in soil water content during the experiment period

**Figure 4:**
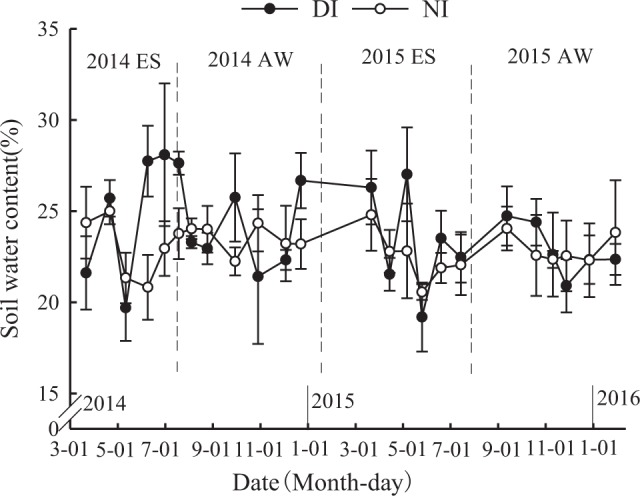
Soil water content (0–20 cm) under the drip irrigation with nutrient solution (DIN) and negative pressure irrigation with nutrient solution (NIN) treatments during the experimental seasons in 2014 and 2015. Error bars show ± standard error of the mean (*n* = 3).

Soil water content in the 0–20 cm layers was exhibited during the four growing seasons in 2014–2015 (Fig. 4). The variation of soil water content was small during in the AW season, with a range of 21.4%–26.7% and 20.9%–24.7% in the 2014AW and 2015AW seasons, respectively. This was lower than the ranges of 19.7%–28.1% and 19.2%–27.0% in the 2014ES and 2015ES seasons, respectively. This may be attributed to lower temperature in the AW season than in the ES season (Fig. 3). A large variation in soil moisture was observed at 0–20 cm soil depth for the DIN treatment (19.2%–28.1% in the two years), which was larger than that of the NIN treatment (20.6%–25.0%), indicating that the NIN treatment can maintain a stable soil water supply. Average soil water content in the 0–20 cm layer over the four growing seasons was higher in the DIN treatment (23.8%) than the NIN treatment (23.0%), but no significant difference was found between the two treatments.

### Changes in soil NO_3_^−^-N content during the experiment period

**Figure 5:**
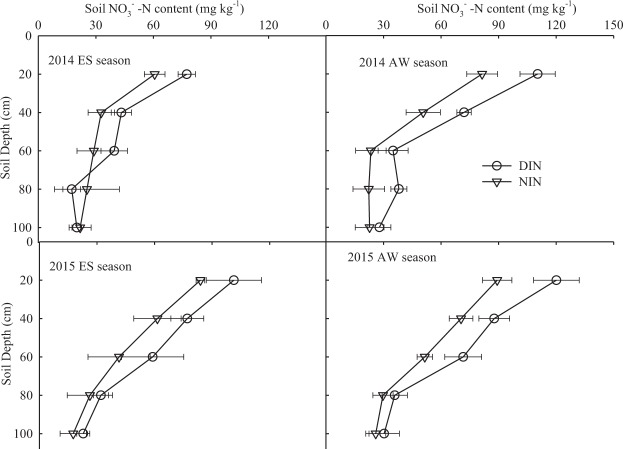
NO_3_^−^-N content in 0–100 cm soil layers after the tomato harvest in the 2014ES season, the tomato harvest in the 2014AW season, the cucumber harvest in the 2015ES season and the tomato harvest in the 2015AW season. DIN, drip irrigation with nutrient solution; NIN, negative pressure irrigation with nutrient solution. Error bars show ± standard error of the mean (*n* = 3).

A similar variation in nitrate distribution along the soil profile was demonstrated at the end of the four growing season in two years of continuous cultivation (Fig. 5). The NO_3_^−^-N content decreased in the 0–100 cm soil profile as the soil depth increased and there were significant differences (P < 0.05) between the 0–40 cm and 60–100 cm soil layers in the four growing seasons. This implies that NO_3_^−^-N was mainly distributed in the 0–40 cm layer. Nitrate storage in the soil profile enhanced with increasing cultivation season, i.e., soil NO_3_^−^-N content of DIN and NIN in the 0–20 cm layer was 77.4 and 60.7 mg kg^−1^ after harvesting in 2014ES season, respectively. These values significantly increased to 120.1 and 95.6 mg kg^−1^ after harvesting in 2015 AW season, respectively. This was attributed to the continuous supply of nutrient solution in the DIN and NIN treatments.

The NO_3_^−^-N content in the 0–60 cm soil layers under the NIN treatment was significantly lower than that under the DIN treatment after the tomato was harvested in the 2015AW season, with decreases of 25.7%, 19.7% and 28.0% in the 0–20 cm, 20–40 cm and 40–60 cm layers, respectively. In the 80–100 cm soil layers, no significant difference was observed between DIN and NIN treatments in the four growing seasons. This indicates that NIN could reduce the residual nitrate in the soil profile compared with DIN, especially for the 0–60 cm soil layers.

### Economic yield and irrigation water and fertilizer use efficiency

The ET, economic yield, irrigation water use efficiency (WUE_i_) and partial factor productivity of fertilizer (PFP_f_) were recorded between DIN and NIN treatments for the four growing cycles from 2014 to 2015 (Table 2). Higher yield can be achieved in the NIN than DIN treatment, but there were no significant differences in economic yields in the four continuous growing seasons. The evapotranspiration (ET) was significantly higher in the DIN than NIN treatment in the AW season, with no significant difference in the ES season. Higher WUE_i_ and PFP_f_ values were observed under NIN than under DIN, although the increase was not significant in the 2014 ES season, the increase was significant in the following three growing seasons. For the NIN treatment, the two-year average WUE_i_ and PFP_f_ over the four growing seasons were significantly higher than those of DIN, with increases of 26.2% and 25.7% (P < 0.05), respectively. Our results indicated that NIN treatment could maintain a high fruit yield and significantly increase WUE_i_ and PFP_f_.

**Table 2:** ET, economic yield, irrigation water use efficiency (WUE_i_) and partial factor productivity of fertilizer (PFP_f_).

Cropping season	Treatment	ET (mm)	Economic yield (t ha^−1^)	*WUE*_i_ (t ha^−1^ mm^−1^)	*PEP*_f_ (kg kg^−1^)
2014 ES	DIN	319.9a	94.0a	0.279a	77.5a
	NIN	295.1a	95.5a	0.320a	88.8a
2014 AW	DIN	270.5a	63.9a	0.225b	62.6b
	NIN	224.1b	69.1a	0.321a	89.3a
2015 ES	DIN	296.2a	71.2a	0.231b	78.1b
	NIN	271.4a	72.6a	0.278a	93.6a
2015 AW	DIN	284.3a	68.2a	0.237b	65.8b
	NIN	238.7b	70.4a	0.308a	85.3a

Mean Values in the same column with different letters in each growth season are significantly different (*n* = 3; P < 0.05).

## Discussion

### Surface soil moisture variation

The water in the surface soil (0–20 cm) had a highly dynamic variation during the different growing seasons due to the high air temperature inside the greenhouse, and temperature had a different variation amplitude in different growing seasons (higher mean daily temperature in the ES season than in the AW season). This may also explain the larger fluctuation in soil water content of the 0–20 cm layer in the ES season than in the AW season. It has been suggested that higher temperature in the greenhouse could promote crop growth and increase the water consumption^16^, which could cause soil moisture variation under different growing seasons.

In the current study, the seasonal variation of soil water content in the 0–20 cm layer for the NIN treatment was 20.6%–25.0% during the whole experiment period, and the average soil moisture over the four growing seasons was 23.0% when a water supply suction of −5 kPa was applied. This was consistent with the results of Li *et al*.^21^ study on cucumber in greenhouse, and who found that the soil water content under irrigation pressure of −5 kPa was maintained at 22.7%. In the current study, the DIN treatment had a larger seasonal variation of soil water content in the 0–20 cm layer than the NIN treatment, with a range of 19.2%–28.1% and 20.6%–25.0% for the DIN and NIN treatments in the two years, respectively. Unlike DIN, NIN is a subsurface irrigation type in which water is supplied under negative pressure directly to the crop root zone, and the water is mainly consumed by the demand of crop growth^19,23^. Thus, the NIN treatment could maintain a more stable soil water supply (about 87% of field capacity) and reduce the amplitude of soil moisture in the 0–20 cm layer compared with the DIN treatment. Zhao *et al*.^22^ also reported that soil water content in the 0–20 cm layer could be maintained at 22.4% (about 89% of field capacity) when water pressures was −5 kPa. On the other hand, the relatively lower fluctuation of soil water under NIN was due to lower irrigated amount compared with DIN treatment, i.e., the annual irrigation amount from 2014 to 2015 for NIN treatment were 513.6 mm and 491.4 mm, respectively, and it reduced by 17.2% and 17.6% than those of DIN treatment, respectively. Panigrahi and Srivastava^27^ showed that surface soil water variation was affected by frequent water application, and soil water content at 0.30 m depth increased with increasing irrigation level.

### NO_3_^−^-N content variation in the soil profile

Very few studies have addressed the impact of negative pressure water supply on N distribution along the soil profile in tomato or cucumber in greenhouses, because studies of negative pressure irrigation have mainly focused on potted cultivation^21,23^. Li *et al*.^21^ found that the negatively pressurized irrigation system could strongly increase distribution uniformity of soil N within the 0–25 cm soil layer compared with conventional irrigation. For the DIN and NIN treatments, great variations in NO_3_^−^-N distribution along the soil profile from 0–100 cm were found in the present study after the tomato or cucumber were harvested in the four seasons (Fig. 5), and the soil NO_3_^−^-N content in the 0–20 cm and 20–40 cm layers was significantly higher than in other soil layers. These results are also in agreement with those of Zhang *et al*.^11^, who found that NO_3_^−^-N was mainly distributed in the top layer of soil.

Our results indicated that the soil NO_3_^−^-N content was mainly distributed in the 0–40 cm soil layer and the lower soil NO_3_^−^-N content under NIN compared with DIN was due to the lower amount of N application. For the NIN treatment, the rates of N application decreased by 24.3% in 2015AW, and soil NO_3_^−^-N content in the 0–40 cm layers was significantly decreased by 19.7%–25.7% compared with the DIN treatment. Previous studies also have observed that a reduced N input could reduce residual N^6,11,28^. Moreover, the irrigation emitter of the NIN treatment was located at 25 cm soil depth and thus the water and nutrients were precisely applied to crop roots, the supply intensity of which would mainly depend on crop requirements. Thus, NO_3_^−^-N content in the soil profile under NIN was lower and more stable than that under DIN. However, with the increase of cultivation duration, the soil NO_3_^−^-N level showed a gradually increasing trend in the 0–100 cm soil layer, especially in the 0–60 cm layer. This may be because of the continuous N input, which can cause residual nitrate in soils. Sun *et al*.^29^ reported that the optimal fertilizer N application rates for greenhouse tomato were 150 kg ha^−1^ and 250 kg ha^−1^ in the autumn-winter and spring-summer seasons, respectively. In the present study, the fertilizer input in NIN treatment was higher than those applied in Sun *et al*.^29^ study, indicating that it might still be potential for further reducing N input for NIN treatment.

### Economic yield and irrigation water and fertilizer use efficiency

Many studies have shown that drip irrigation can reduce water and fertilizer input, and increases fruit yield in greenhouse compared to conventional with irrigation mode^12,30^. In the present study, higher economic yield was observed in the NIN treatment, but there were no significant differences compared with the DIN treatment. We also found that decreasing the amount of nutrient solution by 17.7% (mean value of two years) in the NIN treatment did not result in economic yield losses compared with DIN. Similar results have been obtained by Li *et al*.^24^, who showed that negative pressure irrigation not only significantly reduced water input in a greenhouse, but also increased the tomato yield and improved fruit quality compared with flooding irrigation. The more stable water and nutrient supply under NIN than under DIN may be the major reason for the improvements to tomato and cucumber fruit development. Liang *et al*.^31^ and Farneselli *et al*.^32^ also reported that maintenance of a relatively stable nutrient supply in the root zone was necessary to promote crop growth and increase fruit yield in the greenhouse. For the DIN treatment, higher water and nutrient input did not increase economic yield because they were supplied in excess of greenhouse tomato and cucumber growth requirement. Du *et al*.^3^ showed an optimal irrigation rate of about 223 mm and the optimal fertilizer application rate of about 250 kg N hm^−2^ for tomato grown under drip irrigation in a greenhouse. Zhang *et al*.^33^ suggested that the optimum irrigation level for cucumber under subsurface drip irrigation in a greenhouse is about 262 mm (80% of the evaporation from a standard pan). The irrigation rate and fertilizer input in the DIN treatment of the present study were higher than those applied in previous studies^29,34,35^. Excess water and fertilizer application could affect root growth and function due to inadequate oxygen in the soil, which could cause stomatal closure and losses of N from the profile due to denitrification^36^.

The WUE_i_ and PFP_f_ were significantly higher under the NIN treatment than under the DIN treatment except in the 2014ES season. This was because of the lower nutrient solution input and higher economic yield in the NIN treatment than in the DIN treatment. On the one hand, Negative pressure irrigation is a subsurface irrigation technology, and its irrigation emitter is located at 25 cm soil depth to maintain a small wetted soil zone sufficient for crop water uptake, which could reduce soil moisture evaporation and nutrient deep percolation^23^. Colak *et al*.^37^ reported that subsurface irrigation can provide a stable soil water and nutrient environment for optimal crop growth, improves soil water and nutrient use efficiency and increases yield^33^. On the other hand, NIN treatment had a higher frequency fertigation because the water and nutrients were absorbed actively by the crop root, and surface (0–20 cm) soil moisture and nutrient content remained relatively constant compared with the DIN treatment. Previous studies also reported that high-frequency irrigation is conducive to the maintenance of favorable soil water and nutrient status in the root zone, which can improve crop performance and increase fruit yield^32,36^. Furthermore, the soil water and nutrient level is suitable for crops, because of the frequent fertigation interval under negative pressure irrigation conditions, and the crops do not spend much energy while taking water and nutrient from the soil. They spend most of their energies for growth, development, productivity and increasing fruit quality. Therefore, the WUE_i_ and PFP_f_ were significantly higher under the NIN treatment than under the DIN treatment.

## Conclusions

Soil moisture in the 0–20 cm soil layer under the NIN treatment had a lower variation amplitude (20.6%–25.0%) than under the DIN treatment (19.2%–28.1%), and average soil water content in the NIN treatment during the four growing seasons over the two years was maintained at 23.0% (87% of field capacity). For NIN and DIN, the soil NO_3_^−^-N content was mainly distributed in the 0–40 cm soil layer and showed a gradually increasing trend as the cultivation period increased. The NO_3_^−^-N content in the soil profile of the NIN treatment was significantly decreased and more stable than the DIN treatment, with decreases of 25.7%, 19.7% and 28.0% in the 0–20 cm, 20–40 cm and 40–60 cm layers after the tomato was harvested in the 2015AW season, respectively. Higher economic yields were observed in the four growing seasons under the NIN treatment compared with the DIN treatment. Furthermore, WUE_i_ and PFP_f_ of NIN were significantly increased by 26.2% and 25.7% (P < 0.05) compared with DIN, respectively. Our results demonstrate that negative pressure water supply creates a much more stable soil moisture and soil NO_3_^−^-N content than drip irrigation, leading to increased fruit yield and greatly improved both WUE_i_ and PFP_f_.
